# Exploring expectations of Chinese patients for total knee arthroplasty: once the medicine is taken, the symptoms vanish

**DOI:** 10.1186/s12891-023-06251-x

**Published:** 2023-03-02

**Authors:** Lin Yang, Zhi-Ying Yang, Hui-Wu Li, Yi-Min Xu, Wei-Wei Bian, Hong Ruan

**Affiliations:** 1 Department of Nursing, Shanghai Ninth People’s Hospital, Shanghai JiaoTong University School of Medicine, Shanghai, China; 2 School of Nursing, Shanghai JiaoTong University, Shanghai, China; 3 Department of Orthopedic, Shanghai Ninth People’s Hospital, Shanghai JiaoTong University School of Medicine, Shanghai, China

**Keywords:** Total knee arthroplasty, Preoperative expectation, HSS-TKRES, Mixed methods, Interview

## Abstract

**Background:**

Preoperative expectations of total knee arthroplasty (TKA) outcomes are important determinants of patient satisfaction. However, expectations of patients in different countries are affected by cultural background. The general goal of this study was to describe Chinese TKA patients’ expectations.

**Methods:**

Patients scheduled for TKA were recruited in a quantitative study(*n* = 198). The Hospital for Special Surgery Total Knee Replacement Expectations Survey Questionnaire was used for survey TKA patients’ expectations. Descriptive phenomenological design was used for the qualitative research. Semi-structured interviews were conducted with 15 TKA patients. Colaizzi’s method was used for interview data analysis.

**Results:**

The mean expectation score of Chinese TKA patients was 89.17 points. The 4 highest score items were walk short distance, remove the need for walker, relieve pain and make knee or leg straight. The 2 lowest score items were employed for monetary reimbursement and sexual activity. Five main themes and 12 sub-themes emerged from the interview data, including multiple factors raised expectations, expectations of physical comfort, expect various activities back to normal, hope for a long joint lifespan, and expect a better mood.

**Conclusions:**

Chinese TKA patients reported a relatively high level of expectations, and differences across cultures result in different expectation points than other national populations, requiring adjustment of items when using assessment tools across cultures. Strategies for expectation management should be further developed.

**Level of evidence:**

Level IV.

**Supplementary Information:**

The online version contains supplementary material available at 10.1186/s12891-023-06251-x.

## Background

Knee osteoarthritis (OA) is a degenerative disease affecting older adults. Patients with KOA experience pain, stiffness, and loss of joint function. Total knee arthroplasty (TKA) is the ultimate option for patients with knee OA who have failed to reduce pain or improve joint function after conservative treatment [1]. However, it has been reported that nearly 20% of patients experience dissatisfaction with the TKA outcomes [2]. Numerous studies have attempted to identify factors driving this high dissatisfaction rate. There is growing evidence that unfulfilled preoperative expectations are important determinants of patients’ dissatisfaction with TKA [3–5].

Previous studies have indicated that patients generally have high and unrealistic expectations of TKA [6]. 85% of patients expect to be pain-free, and 52% expect to be no functional limitations after TKA, but only 43 and 20% patients achieve these expectations at 2 years after TKA, respectively [7]. Patients’ expectations also higher than physicians’ expectations [8]. Therefore, it is important to pay attention to preoperative expectations of TKA patients.

Mancuso et al. [9] conducted a study to assess preoperative expectations of TKA patients, known as the Total Knee Replacement Expectations Survey in Hospitals for Special Surgery (HSS-TKRES). The questionnaire used in this survey has been translated and applied by researchers in many countries. The Chinese version of the questionnaire also shown good reliability and validity in the Chinese TKA population [10]. However, studies in different countries have found that while the questionnaire contains the expectations of most patients, there are some that do not. Researchers in the UK added items such as “pain that interferes with sleep” when using the HSS-TKRES questionnaire, and approximately 70% of patients rated this expectation as very significant [11]. Canadian researchers compared open-ended expectations questions with HSS-TKRES questionnaire and found that the additional items included better sleep, walking without a limp, self-care, less or no pain medication, weight loss, and ability to sit or stand for long periods [12]. Therefore, with the improvement of surgical technology and people’s demand for quality of life, it is worth exploring whether this questionnaire item can cover the current preoperative needs of Chinese TKA patients.

Additionally, more studies indicated that differences in expectations of TKA patients are not only due to sociodemographic or disease factors [12, 13], but also the patients’ expectations can vary significantly across countries or cultures. It is found that Australian patients have different expectations regarding walking ability and walking aid demand from the UK and the United States (US) [13]. Another research suggested that patients in the US and Canada had significantly different expectations for specific types of exercise [12]. These differences in expectations could be explained by the fact that people in different countries have various cultural, lifestyle and social background.

Unlike the aforementioned Western countries, China is an oriental country with a specific cultural tradition, mainly influenced by Confucian values. Previous studies have shown that Chinese patients with TKA may have different concerns due to the differences in social culture and lifestyle between China and other countries [14]. Their expectations may also be different from the expectations of patients in other countries [15]. Therefore, our hypothesis is that the expectations of Chinese TKA patients are unclear and may exceed the scope of the questionnaire items. So, the primary goal of our research is to understand what the current expectations of Chinese TKA patients are. The secondary goal is to compare the differences between Chinese TKA patients’ expectations and the questionnaire items, and to explain why these expectations arise. We hope these results will help us to improve the instrument for measuring the expectations of Chinese TKA patients, and provide a reference for building the expectations management strategies in the future.

## Materials and methods

This mixed-methods study included a quantitative survey and qualitative research. The study was approved by the Medical Ethics Committee of the Ninth People’s Hospital affiliated to Shanghai Jiaotong University School of Medicine, Shanghai, China (SH9H-2021-T344–2). The quantitative survey was conducted from May to October 2019, and the qualitative interviews were conducted from February to July 2020. Participants were recruited from the orthopedic ward of a tertiary general hospital in Shanghai, China. Patients were included if they were scheduled to undergo primary TKA. Patients who were scheduled for revision TKA, single-compartment knee arthroplasty, staging or bilateral knee arthroplasty were excluded. Patients who were unconscious or critically ill, or who were unable to express themselves clearly, were also excluded. All participants provided written informed consent and were informed that participation was on a voluntary basis and that they could withdraw from the study at any time without explanation. Demographic data and quotations were presented in a way that prevented the identification of individuals. All collected data would be handled confidentially. Participants were informed that data would be analyzed and published in a research journal.

### Quantitative survey

Convenient sampling was used for quantitative surveys. A general information survey and the HSS-TKRES questionnaire were included in the questionnaire. The former included the patient’s age, gender, educational status, work status, living companions, and co-morbidities. The HSS-TKRES questionnaire was used to assess patients’ expectations, including expectations on postoperative pain, physical activity, social activity, and mental health [9]. Chinese researcher Wang et al. [10] translated the tool, adapted for cross-cultural adaptation, and performed validation and reliability tests. Chinese version of the questionnaire changed the original response model as follows,‘back to normal or completely improvement’(4 points), ‘a lot of improvement’(3 points), ‘a moderate amount of improvement’(2 points), ‘a little improvement’(1 points) or ‘this expectation does not apply to me or I do not have this expectation’(0 point). The questionnaire contains 19 items. The total score ranging from 0 to 76 points was adapted into a 100-point scale, with higher scores representing higher expectations.

The primary outcome was the proportion of patients’ expectations listed on the questionnaire. To identify these proportions within an uncertainty of 15%, the 95% confidence intervals for any proportion needed to be ±7.5%. This required 170 patients. Assuming a loss of 10% gave 187 patients. Thus, 206 patients were required to be recruited to the study.

Patients were assessed by the main researcher (YL) on admission for meeting with inclusion and exclusion criteria. The questionnaires were distributed by 5 trained questionnaire collectors. Patients were asked to complete the questionnaires themselves. For those patients who could not read text, collectors assisted them in reading and writing. The questionnaires were collected on the spot.

### Qualitative research

Descriptive phenomenological design was used in order to describe and explain patients’ expectations of TKA [16]. Purpose sampling was used. The principle of maximal heterogeneity of demographic factors was followed in the sampling process [17], accounting for patients’ age, gender, education level, work status, living companions (alone, with parents, couple with children). The sample size was determined according to the principle of theme saturation [18].

Semi-structured interviews with open-ended questions were designed to analyze the preoperative expectations of TKA patients. The first interview with every participant was conducted face-to-face, at the hospital bed-side, in the time ranges of 0 to 2 days before the operation. Because patients’ cognition of the treatment outcome and rehabilitation process before surgery is limited, some expectations may not be considered until they experience it. So, the patients were interviewed again about 1 month after the operation in order to understand the comprehensive expectations of TKA patients. The second interview was conducted with face-to-face when the patient came to the outpatient clinic for follow-up or by telephone call.

The One-to-one interviews were conducted at a time convenient for the participants and lasted 20–40 minutes. The interview schedule was designed based on the previous literature and revised according to results of two pre-interviews, available in the supplemental material. The interviews were conducted by the first author (YL), a PhD student and a female nurse working in orthopedics, trained in qualitative methods and experienced in interviewing. All interviews were audio-recorded and transcribed verbatim in Chinese within 2 days after each interview. Several valuable nonverbal behaviors observed during the interview were also included, such as silence, thinking, hesitation, pausing, and facial expressions. The study followed the Consolidated Criteria for Reporting Qualitative Research (COREQ) checklist.

### Analysis of data

Data from questionnaire results were analyzed with SPSS 21.0 (SPSS Inc., Chicago). The general condition of the included patients was described, including the mean and standard deviation of age, and the number of cases and rates for other variables. The mean and total scores for each item in the HSS-TKRES questionnaire were calculated. The figure showing the score of the questionnaire entries was created using Excel software.

**Fig. 1:**
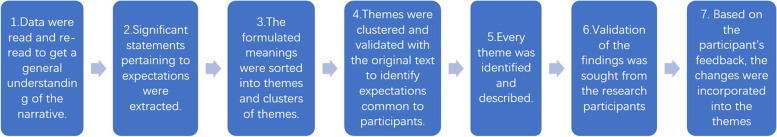
Process of analyzing qualitative research data

Qualitative data were independently analyzed by two researchers (YL and XYM) using NVivo Software (QSR International, Doncaster, Victoria, Australia). Disagreements in the analyses were discussed by the research team and final decisions were made. Data collection and analysis were concurrently conducted to allow for the exploration of the key themes and to evaluate when data saturation occurred. Colaizzi’s method was used for data analysis [19], which helped to gain a better idea of each participant’s experiences. The following data analysis steps were performed (Fig. 1). Quotes used in this report were then translated into English by the main researcher (YL), and revise translations through research group discussions.

## Results

### Quantitative research

**Fig. 2:**
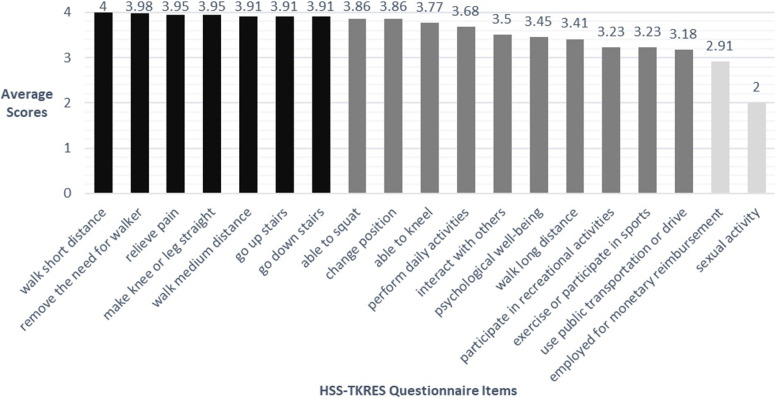
Average score of items in HSS-TKRES questionnaire for Chinese TKA patients, ranked from highest to lowest score. Mark above 3.9 points in black, 3–3.9 points in dark grey, and below 3 points in light grey

A total of 206 questionnaires were sent out, and 198 valid questionnaires were returned. Table 1 contains the demographic, clinical characteristics of the participants and the total scores of the HSS-TKRES questionnaire. The scores of each item in the HSS-TKRES questionnaire of Chinese TKA patients was shown in Fig. 2, listed by item score from left to right.

**Table 1:** Characteristics of quantitative research sample

	Research sample (*n* = 198)
Age, years, scope (mean, SD)	56–94 (72.2, 13.82)
Gender, n (%)	
Male	49(24.75)
Female	149(75.25)
Educational status, n (%)	
Primary school and below	54(27.27)
Junior high school	63(31.82)
Higher education	81(40.91)
Work status, n (%)	
Retired	153 (77.27)
At work	18(9.09)
Unemployed	27(13.64)
Living companions, n (%)	
Live alone	20(10.10)
With partner	81(40.91)
With parent(s)	14(7.07)
With child (ren)	76(38.38)
With partner and child (ren)	7(3.54)
Number of co-morbidities, n (%)	
0	29(14.65)
1	73(36.87)
2	80(40.40)
Over 2	16(8.08)
HSS-TKRES scores, points, scope (mean, SD)	48.68 ~ 100 (89.17, 13.89)

*SD* standard deviation

### Qualitative research

**Fig. 3:**
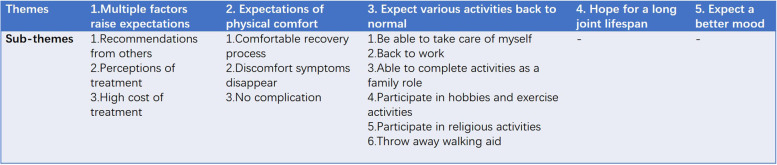
Themes and sub-themes emerged from qualitative research

Demographics of interview sample (*n* = 15) were listed in Table 2. Five main themes and 12 sub-themes emerged from the interview data (Fig. 3).

**Table 2:** Interview sample demographics (*n* = 15)

Unique identifier	Age	Gender	Education level	Work state	Living companions
P1	72	F	High school	Retired	Alone
P2	69	F	Primary	Retired	PA, CO
P3	75	M	Junior high	Retired	CO
P4	79	F	Junior college	Retired	CH
P5	85	M	Junior high	Retired	CO, CH
P6	72	F	Junior high	Retired	CO
P7	62	F	Illiteracy	On work (Farming)	PA, CO
P8	80	F	Junior high	Retired	Alone
P9	74	M	High school	Retired	CO, CH
P10	60	M	Primary	On work (manager)	CO
P11	57	F	Primary	On work (teacher)	PA, CO
P12	66	F	Junior high	Retired	CO, CH
P13	76	F	Primary	Retired	CH
P14	70	F	Illiteracy	Retired	CO
P15	71	M	Junior high	Retired	CO, CH

PA live with parent(s), CO live with couple, CH live with child(ren)

### Theme 1: multiple factors raise expectations

#### Recommendations from others

Some patients’ friends, family members or neighbors who have been treated with TKA have raised patients’ expectations by informing them of the successful outcome of surgery.

*“An aunt in our community underwent surgery here. Now she can walk very well. She introduced me to the surgery. I hope I can obtain the same results as she did.”(P6, pre-op).*

Some patients mentioned that they had read positive reviews online about the TKA procedure and the orthopedic surgeon, so they thought they could have such a good outcome as the patients who gave positive reviews.

*“I read the comments on the Internet saying that he has very good surgical skills and a good attitude to patients, so I was attracted here. I believe he will not have any problems with performing the operation.”(P9, pre-op).*

Some patients are referred to specific physicians because they have been rejected at other hospitals due to complex disease conditions. These patients consider this doctor as their last hope and that he can save them.

*“Other doctors introduced me to this hospital. They said that my situation is complicated and suggested that I come here for surgery.”(P5, pre-op).*

#### Perceptions of treatment

Most patients with knee OA had received other treatments prior to TKA. And when other treatments did not work well, patients obtained information from various sources about TKA treatment being effective. This perception then raises the patient’s expectations about the effectiveness of the treatment.

*“Anyway, it must be better than conservative treatment. Conservative treatment did not delay the pain for a very long time, and the pain cycle was getting shorter and shorter.”(P13, pre-op).*

#### High cost of treatment

Some participants pointed out that the surgery costs a lot, thus presenting a huge financial burden on their families. Undoubtedly, high costs also led patients to have high expectations. This was a value-for-money concept, where something that is high-priced is expected to be good.

*“Expectations must be high. after all, so much money was spent on this operation.”(P7, pre-op).*

### Theme 2: expectations of physical comfort

#### Comfortable recovery process

Participants heard about the postoperative uncomfortable process from ward mates, which increased their expectations and worries, such as fear of pain. In postoperative interview, some patients felt the discomfort during the recovery process were unexpected, surprising and confusing. Many patients mentioned the remarkable pain during postoperative exercise, including pain during hospitalization and home-rehabilitation self-exercise. From interview, patients expressed expectations for a comfortable recovery process.

*“I have seen some patients vomiting after the operation. I hope I will not be so uncomfortable.”(P12, pre-op).*

*“In the hospital, when the doctor bent my knee, it hurt way too much. I never thought it would hurt so much. I really hope that there is no need to exercise.” (P1, post-op).*

#### Discomfort symptoms disappear

All patients reported expectations related to the disappearance of preoperative symptoms, which was the main reason for patients to seek medical treatment. Most patients expected the symptoms to disappear as soon as possible. Some patients understood that it takes time for symptoms to disappear and that postoperative effects may vary from person to person; however, they hoped that they would be the ones to recover quickly. For the prediction of the recovery time, some participants used a Chinese saying “Bone injury needs a hundred days of recovery”.

*“Every morning when I get up, my leg hurts, and I can’t move. I hope it will get better after the operation.”(P8, pre-op).*

*“The effect of a joint replacement is definitely immediate; I estimate that I will recover in about a month.” (P14, pre-op).*

*“I’m usually in good health, better than my peers. I don’t have any illnesses, so I think I will recover faster.” (P2, pre-op).*

#### No complication

Most patients were informed of the risk of postoperative complications such as joint infections, deep vein thrombosis and bleeding prior to surgery. The interviewees were worried and nervous about this, hoping that no complications would occur and that they would get through the treatment phase. During the postoperative interviews, patients were asking and speculating whether some of the symptoms were manifestations of complications, also reflecting patients’ expectations of no complications.

*“I hope it will go smoothly. I hope I don’t get infected or broken joints.”(P2, pre-op).*

*“A few days ago, my leg was a little swollen and painful, I was very worried about a potential blood clot.” (P11, post-op).*

### Theme 3: expect various activities back to normal

#### Be able to take care of myself

Interviewees identified the ability to take care of themselves as the most basic expectation. Especially for patients who live alone, they have higher demands and desires for post-operative mobility because they need to do all the housework and shopping by themselves.

*“I want to be able to run a little bit longer and buy groceries, but I don’t want my knees to hurt when I go far.”(P1, pre-op).*

#### Back to work

In this study, most TKA patients had reached retirement age, but there were patients who were still working or engaged in agricultural activities. They wanted to be able to have the ability to return to work after surgery.

*“I hope I can grow land after the operation.”(P7, pre-op).*

#### Able to complete activities as a family role

Most of the participants live with their children and grandchildren. They expected to be able to resume their family roles after the surgery, such as taking on light household tasks, cooking and taking their grandchildren to school, otherwise they felt guilty for not being able to help their families.

*“Knee problems prevented me from doing many things I want to do at home. I can’t help my family.”(P15, pre-op).*

*“I can’t squat down to give the baby a bath.”(P4, pre-op).*

#### Participate in hobbies and exercise activities

Some interviewees pointed out that the expectation from the operation outcome included the ability to engage in their hobbies and exercise activities after the operation, such as walking, dancing, square dancing, traveling, and shopping.

*“I like traveling very much. After I got sick, my friends stopped asking me to travel farther away. But I will continue to travel after the operation is complete.”(P6, pre-op).*

*“I used to square dance when I was not sick. I hope that after the operation, I can recover and continue dancing.”(P9, pre-op).*

#### Participate in religious activities

One patient declared that she needed to kneel for their religious activities, so she was hoping her new joint would bend well.

*“I believe in Buddhism. I need to kneel down to worship Buddha. I don’t know if I will be able to do this after the operation. I hope I will.” (P13, pre-op).*

#### Throw away walking aid

Use assistive devices before surgery, which makes them feel that they are regarded as “disabled”. Although in the early postoperative period, patients are often advised to use mobility aids to continue to move so, they expect to throw away these aids as soon as possible.

*“After I got sick, I had to use crutches or a wheelchair. I’m embarrassed to go out to meet friends. I’m afraid they will think I’m disabled.”(P3, pre-op).*

*“When can I stop using this walking aid?” (P10, post-op).*

### Theme 4: Hope for a long joint lifespan

Interviewees expressed their expectations for the life span of their joints, hoping to reach the life span they were told by their doctors without the need for revision surgery.

*“I hope this joint can last longer. Hope it won’t break down soon so that I need to operate it again.”(P14, post-op).*

### Theme 5: expect a better mood

Interviewees mentioned that joint disease indirectly led to worsening moods, feelings of disappointment, helplessness, and self-blame. Therefore, they expected to improve not only their physical health, but further their mood as well.

*“When I was sick, I wanted to do a lot of things but couldn’t. At this time, I resented myself very much. Only when my knees get better will my mood improve.”(P1, pre-op).*

## Discussion

Our survey results revealed that the average expectation score of Chinese patients regarding TKA was (89.17 ± 13.89) points, which are relatively high. One of Chinese idioms argues that “Once the medicine is taken, the symptoms vanish”, which is the dream of every patient, but also a stereotype that medical treatment can cure everything. Dutch researchers investigated the expectation of knee surgery doctors on the outcomes of TKA, reporting the score of only (66.0 ± 14.0) [8]. Patients’ expectations are higher than physicians’, indicating that patients’ expectation of outcomes are unrealistic and difficult to achieve with current medical technology [11]. Therefore, based on the results of TKA patient expectation survey, it is necessary to carry out the expectation management to avoid the gap between unrealistic expectations and the reality, which may lead to potential medical disputes. Considering the differences in the technical and skill levels between different hospitals or physicians, the specific content of expectation management needs to be adjusted according to the real situation so it could effectively help patients to make clinical medical decisions.

Pain and joint dysfunction are the main reasons why knee OA patients seek out treatments. In our survey, patients also showed high expectations for these items, which is consistent with findings from other countries. Differently, our study found that Chinese patients had extremely high expectations regarding the “removal of the need for walker”, whereas this expectation ranked only 14–16 in studies from Australia [20], Belgian [21], and South Korea [22]. This large variation in expectation may be due to the social and cultural differences across different countries. The analysis of qualitative interviews validated the results of the survey and explained the reasons for this phenomenon. Regardless of before or after surgery, Chinese patients tend to dislike using any walking aid because of negative impact on their personal image and social activities. Walking aid is an assistive technology devices (ATD), which is considered as an influential factor that identifies or “marks” someone as having a disability [23]. Public misunderstanding about the use of ATD labels TKA patients as “disabled”, which may affect an individual’s mental health, causing negative attitudes and behaviors [24]. Social stereotypes about “disability” also may induce feelings of stigma and discrimination [23]. In addition, restrictions on barrier-free facilities have brought some inconveniences to go out, affecting the quality of life [25]. For example, China’s buses are rarely equipped with bus wheelchair ramps or lift devices, which make it hard for wheelchair-bound patients to take bus, thus reducing their opportunities to participate in social activities. Consequently, these social backgrounds and facility characteristics have made Chinese patients to have extremely high expectations for eliminating the need for a walker. This suggests that in managing patients’ expectations, providers can inform them of the average time they will need a walker after surgery, set “ removal of the need for walker “ as a goal for rehabilitation, and encourage patients to take active rehabilitation exercises to achieve these expectations as soon as possible.

Another characteristic of Chinese patient expectations is high expectations for the ability to kneel, squat, and engage in other high flexion movements, which are relatively higher than that expected in patients from other countries [26]. Combined with the results from qualitative research, it can be concluded that some Chinese habits and peculiar behaviors require kneeling or squatting [27], such as religious activities (bow down to the Buddha), worship ancestors, and squatting during defecation and washing things [28]. Chinese patients expect to have the ability to continue these habitual behaviors after TKA. Yet, a previous study showed that 68% of patients could not kneel until 18–24 months after TKA [29]. The top 2 unsatisfactory items for TKA patients were sitting with legs crossed and squatting [30]. From the above, it is not difficult to find that complete high flexion in the early postoperative period is a great challenge. Considering this, it is very important to assess patients’ expectations of high flexion movements before surgery. After discussing and adjusting the expectations, the doctor and the patient make a joint decision together on the choice of prosthesis and the surgical plan. For those patients with high expectations for kneeling and squatting, a single high-flexion posterior stabilized fixed-bearing prosthesis or a postoperative kneeling rehabilitation protocol should be considered to achieve their expectations [29, 31].

Chinese patients reported low expectations of improved sexual performance in this study, which is in agreement with results from other countries [21, 26, 32]. TKA patients were mostly elderly. The relatively low sexual desire in this age group may have contributed to the decreased expectations. It is found that patients experienced sexual limitation for an average of 17.1 months before surgery because of pain and diminished range of motion or flexibility. Postoperatively, patients resumed sexual activity after an average of 2.4 months, while 25% of patients had reduced sexual frequency in the first year after surgery [33]. Only 31.18% of patients achieved the expected sexual activity 1 year after surgery [34]. Thus, patients’ expectations of sexual ability might be reasonable that TKA cannot eliminate sexual limitations, but gives patients more liberty in selecting their sexual positions [33].

The survey of the HSS-TKRES in the Chinese population suggests that the instrument already reflects part of the patients’ expectations. Yet, the results of the qualitative research were very valuable as they revealed that Chinese patients still have specific expectations that were not presented in the questionnaire. For example, comfortable recovery process, no complications, able to complete activities as a family role, and hope for a long joint lifespan. Therefore, there is a necessity to enhance the tool for surveying the expectations of Chinese TKA patients. The following discussion focuses on these expectations beyond the questionnaire items.

The qualitative research results gave us a new understanding of the expectations of TKA outcomes: the expectation is not only the result, but also the process. During the interview, many patients mentioned their expectations about a comfortable recovery process. A previous study found that patients’ perception of surgery failure was related to the outcome failure and the functional failure [35]. Specifically, it includes refractory index joint pain, the occurrence of postoperative adverse events, inability to resume normal activities or go back to work, little or no improvement in quality of life, and early revision surgery [35]. It has been concluded that it is not sufficient to only focus on the final outcome, and that nuisance symptoms during recovery also negatively affect satisfaction and even lead to decision regret [36]. These experiences of postoperative discomfort are what we need to be concerned about and find solutions to alleviate them. However, before completely solving these problems, patients should be aware of the discomfort they might go through after surgery to reduce anxiety. Healthcare providers should also provide intervention suggestions for uncomfortable symptoms to reduce discomfort during recovery.

In the preoperative and postoperative interviews with patients, they strongly expressed the expectation that no postoperative complication. Although this item was not reflected in the HSS-TKRES questionnaire, it is not necessary to investigate patients’ expectations of complications because no one could possibly hope for that. However, it is an important element in expectation management. The occurrence of postoperative complications can affect the postoperative recovery process and even the outcome, potentially causing great suffering and financial burden to the patient. Therefore, information on indispensable complications should be included when performing patient expectation management, which can be an important element in influencing patient decisions.

Patients reported their expectations about able to complete activities as a family role after TKA, which is one of the distinctive expectations in Chinese population. Different from most Western countries, China is a family-cored culture where filial piety has great importance. It is a very common phenomenon that elderly Chinese live with their children, even with grandchildren. The elderly received care from their children but were also required to provide household support and inter-generational care for their children [37]. In China, 58% of grandparents provide childcare to their grandchildren, compared to only 6% in South Korea and lower than 10% in most European countries and the US [38, 39]. Nevertheless, elderly living in three-generation households experiences a slightly more rapid health decline than those who live alone [40]. Therefore, these people may have higher expectations of resuming their previous roles after surgery. In this case, it is suggested to split the task list for achieving family roles into functional requirements for joint activities and discuss this issue with both patients and family members. The time required for recovery should also be communicated so that family members can be adequately prepared and make corresponding adjustments in their living arrangements.

Qualitative interviews also revealed that the lifespan of joints was of great concern for patients. The lifespan of one’s joint is one of the long-term outcomes of the treatment, which is difficult to answer accurately. This issue is characterized by uncertainty and affected by many factors; yet, most patients do wish to know [41]. Accordingly, we suggest providing patients with both the average lifespan of a joint and the factors influencing it, introducing the importance of postoperative rehabilitation to patients and jointly working to improve joint life span.

Another issue of concern is that in the previous preoperative expectations survey, we did not define how long after surgery is it to meet these expectations. In the interviews, patients mentioned their expectations about the recovery period from immediate to 3 months. However, it may take about 1 year of rehabilitation activities after surgery with some discomfort during the period. Expectation fulfillment following TKA changes with time [42]. TKA patients who felt unsatisfied at early follow-up expressed satisfaction at late follow-up [43]. Thus, it is crucial that time should not be excluded from consideration when discussing expectations of surgical outcomes. In the HSS-TKRES questionnaire, the original authors gave some examples of definitions of the items to unify patients’ understanding of the concepts. We should also learn from this successful experience when developing the questionnaire for the Chinese TKA patients by defining the concepts for newly added expectations.

## Study limitations

This study had certain limitations. First, the study only recruited patients from a Grade-A general hospital in Shanghai, and may not be representative of the overall Chinese population. Nevertheless, patients from across the country ensured the richness of qualitative research data sources. Then, the main researcher had analyzed the results of the survey before the qualitative research, which may affect the interpretation of the interview data. But given that the main researcher had received systematic qualitative research training, this bias was minimized by using suspension in the analysis process and returning the results to the interviewees for verification. Last, Chinese qualitative interview data may have differences in semantic interpretation during translation into English. We made modifications through group discussions to remedy these deficiencies.

## Conclusion

Our research found that Chinese patients have high expectations of TKA outcomes, with some expectations not included in the HSS-TKRES entries due to Chinese cultural, social, and people activity characteristics. In addition, the patient’s expectations for a comfortable postoperative recovery process, length of recovery, joint lifespan, and no complication should also be of concern to providers. Tools for measurement should be improved and strategies for expectation management should be developed further.

## Supplementary Information

**Additional file 1.**

## Data Availability

The datasets generated and/or analysed during the current study are not publicly available due to participant confidentiality, but are available from the corresponding author on reasonable request.

## Abbreviations

OA: Osteoarthritis
TKA: Total Knee Arthroplasty
HSS-TKRES: the Hospital for Special Surgery Total Knee Replacement Expectations Survey
UK: United Kingdom
US: United States
ATD: Assistive Technology Devices
