# Supplementary material for: Exploring expectations of Chinese patients for total knee arthroplasty: once the medicine is taken, the symptoms vanish
Source: BMC Musculoskelet Disord. 2023 Mar 2;24:159. doi: 10.1186/s12891-023-06251-x (PMC9979482; doi:10.1186/s12891-023-06251-x)
Supplement: Supplementary file 1 — Additional file 1. [file 12891_2023_6251_MOESM1_ESM.docx]

**Interview schedule**

Preoperative interview

1. What are your expectations of knee replacement surgery?
2. What do you hope to achieve after the operation? (at different time points)
3. Why is there such an expectation?
4. Where did you learn about the possible results of this surgery?

Postoperative interview

1. After going through the surgery and recovery, what are your expectations for knee replacement surgery at different points? Compared with preoperative expectations, is there anything to add?
2. Why is there such an expectation?
